# Whole-Genome Sequence of Brucella melitensis Strain B7, Isolated from a Blood Sample of a Brucellosis Patient from Hulunbuir, Inner Mongolia, China

**DOI:** 10.1128/MRA.00119-19

**Published:** 2019-06-13

**Authors:** Guoqing Zhang, Chen Liang, Chenfang Liu, Jing Zhang, Xiaoyu Pi, Yujia Zhang, Xiuwen Liang, Lijun Wang, Beiwen Zheng

**Affiliations:** a Department of Brucellosis, Hulunbuir People’s Hospital, Hulunbuir, China; b School of Medicine, Inner Mongolia University for Nationalities, Hulunbuir, China; c Collaborative Innovation Center for Diagnosis and Treatment of Infectious Diseases, State Key Laboratory for Diagnosis and Treatment of Infectious Diseases, the First Affiliated Hospital, College of Medicine, Zhejiang University, Hangzhou, China; University of Delaware

## Abstract

We report here the draft genome sequence of Brucella melitensis strain B7, isolated from a blood sample of a brucellosis patient from Hulunbuir, Inner Mongolia, China. This report describes the genome sequence of a human B. melitensis isolate, which is endemic in this area of China.

## ANNOUNCEMENT

*Brucella* is a genus of Gram-negative facultative intracellular bacteria that cause brucellosis in many animals and is also an important pathogen responsible for human disease (1). Globally, brucellosis is one of the most common zoonotic infections, with an incidence estimated at 500,000 new cases per year (2). Human brucellosis is mainly transmitted from its animal reservoirs to humans by direct contact with infected animals or through raw meat and dairy products (3). In China, Brucella melitensis has widespread geographical distribution and has caused outbreaks and sporadic cases in past decades (4–7). It is worthy to note that the Inner Mongolia Autonomous Region is the most severe endemic focus of human brucellosis in China (4, 8, 9). Here, we present the whole-genome sequence of B. melitensis strain B7, isolated from a human brucellosis case from Hulunbuir, Inner Mongolia, China.

B. melitensis strain B7 was isolated from the blood sample of a 67-year-old patient with brucellosis admitted to the Department of Brucellosis, Hulunbuir People’s Hospital. The molecular manipulations of B. melitensis strain B7 were carried out in a biosafety level 3 laboratory facility. Bacteria were cultured on brucella agar medium (BD BBL, USA) at 37°C for 48 h. High-molecular-weight genomic DNA was extracted using a bacterial DNA kit (Omega Bio-tek, Norcross, GA, USA). The concentration of genomic DNA was determined using a Qubit 2.0 fluorometer and Qubit double-stranded DNA (dsDNA) high-sensitivity (HS) assay kit (Invitrogen, Life Technologies, USA). An aliquot of the genomic DNA was subjected to analysis using a bioanalyzer (Agilent Technologies, USA), and we confirmed that no degradation had occurred. The sequencing library was prepared using the Nextera mate pair library preparation kit (Illumina, San Diego, CA, USA). Whole-genome shotgun sequencing was performed by using an Illumina NovaSeq 6000 genomic sequencer at Novogene Co. (Beijing, China), with a 2 × 150-bp paired-end sequencing strategy. The raw reads were filtered for low-quality reads and adaptor trimmed using Trimmomatic version 0.36 with the default settings (10). All clean reads were assembled into scaffolds using Velvet version 1.2.07 (11), and PAGIT flow was used to prolong the initial contigs and correct sequencing errors (12). The quality of the genome assemblies was determined using QUAST version 5.0.0 (13) by comparing the contigs to a previously published B. melitensis genome sequence (GenBank accession number NC_003317). Genomic sequences were annotated using the RAST server 2.0 (14). The tRNAs and rRNAs were identified using tRNAscan-SE version 1.21 (15), RNAmmer (16), and Rfam database (17). The resistome analysis was carried out using the Comprehensive Antibiotic Resistance Database (CARD) (https://card.mcmaster.ca/). The origins of replication for the plasmid were determined by Plasmid Finder 2.0 (https://cge.cbs.dtu.dk/services/PlasmidFinder/), insertion sequence (IS) elements and transposons were identified using ISfinder (http://www-is.biotoul.fr/), prophages were predicted by PHAST (18), and CRISPR arrays were identified using CRISPR finder (http://crispr.i2bc.paris-saclay.fr/). All of the programs were used with default settings.

A total of 3,768,556 raw reads (339.8× depth of coverage) were generated from the sequencing run. The assembled genome sequence of B. melitensis strain B7 was found to be 3,308,574 bp in length, with an *N*_50_ value of 2,168,882 bp. The genome consists of two circular chromosomes, with 2,168,792 bp in chromosome I and 1,139,782 bp in chromosome II (Table 1). The G+C contents of chromosome I and chromosome II are 57.1% and 57.5%, respectively. About 87.03% (2,879,377 bp) of the nucleotide sequences are predicted to be coding sequences. These contigs contain 3,153 coding sequences (CDSs), 54 tRNAs, and 9 rRNAs. Moreover, this strain has 157 noncoding RNAs (ncRNAs) and 1 transfer-messenger RNA (tmRNA). An analysis of IS elements indicated that B7 possesses at least 256 putative ISs.

**TABLE 1:** General characteristics of the Brucella melitensis strain B7 genome

Genomic characteristic	Value for:	
	Chromosome I	Chromosome II
Size (bp)	2,168,792	1,139,782
Coding regions (%)	87.2	86.7
G+C content (%)	57.1	57.5
No. of pseudogenes	31	19
No. of protein-coding sequences	2,072	1,081
No. of rRNAs	6	3
No. of tRNAs	39	16
No. of other RNAs	99	59
No. of ISs	101	155
No. of prophages	1	0
No. of CRISPR arrays	1	0

To the best of our knowledge, the genome sequence of a human B. melitensis isolate from Inner Mongolia has not been previously described, even though B. melitensis infection is endemic in this area of China. In this study, we described the genome sequence of B. melitensis strain B7 causing spondylodiscitis, which is the foremost cause of the debilitating and disabling complications of brucellosis (19). Further studies are required to include more clinical isolates from Inner Mongolia for whole-genome sequencing and comparison with selected sequences of B. melitensis available from NCBI.

### Data availability.

This whole-genome shotgun project has been deposited at DDBJ/EMBL/GenBank under the accession number RWIG00000000. The version described in this paper is the first version, RWIG01000000. Raw reads are available in the NCBI SRA under accession number PRJNA508265.
